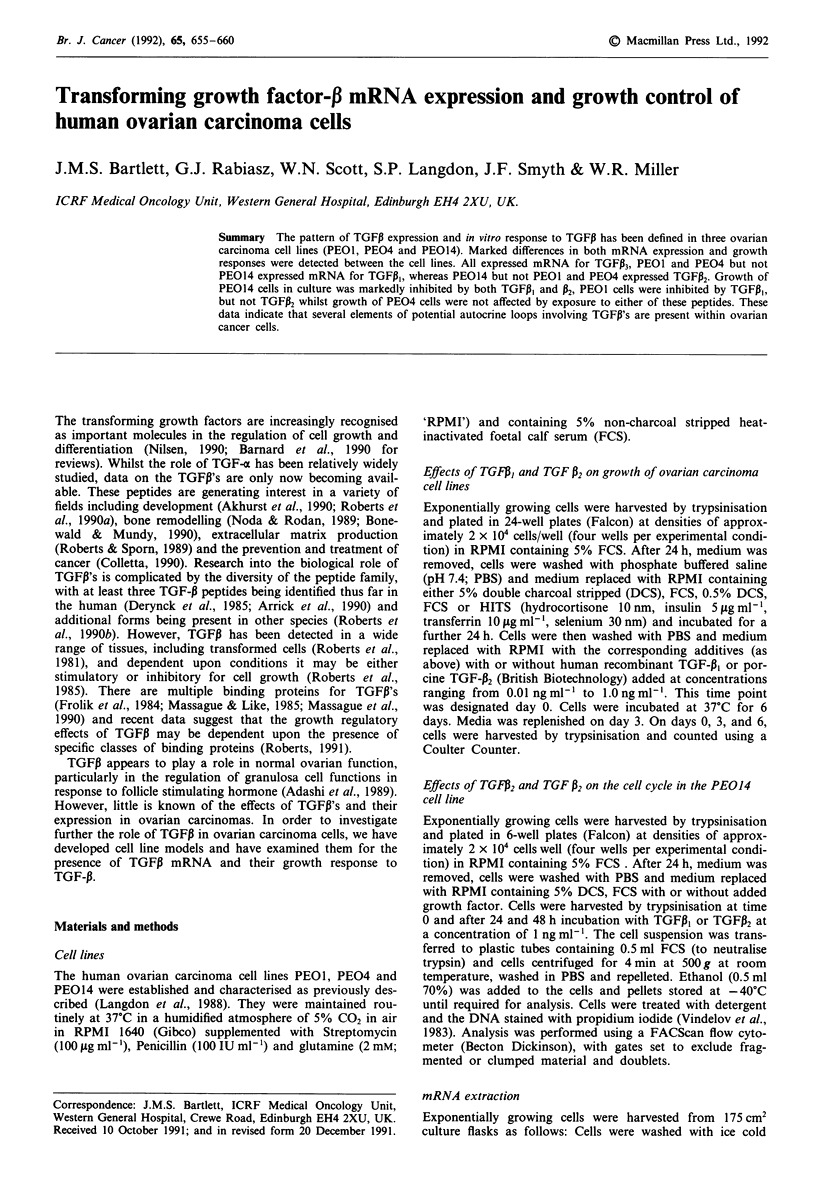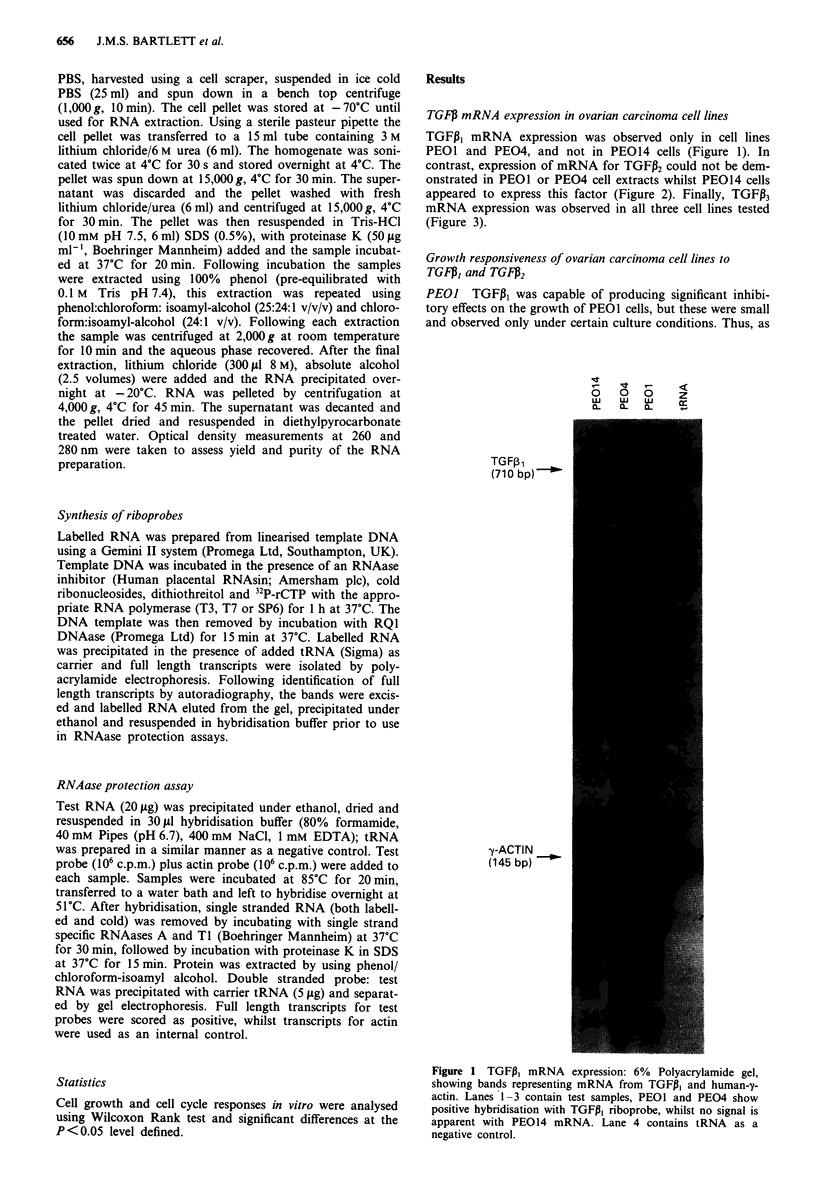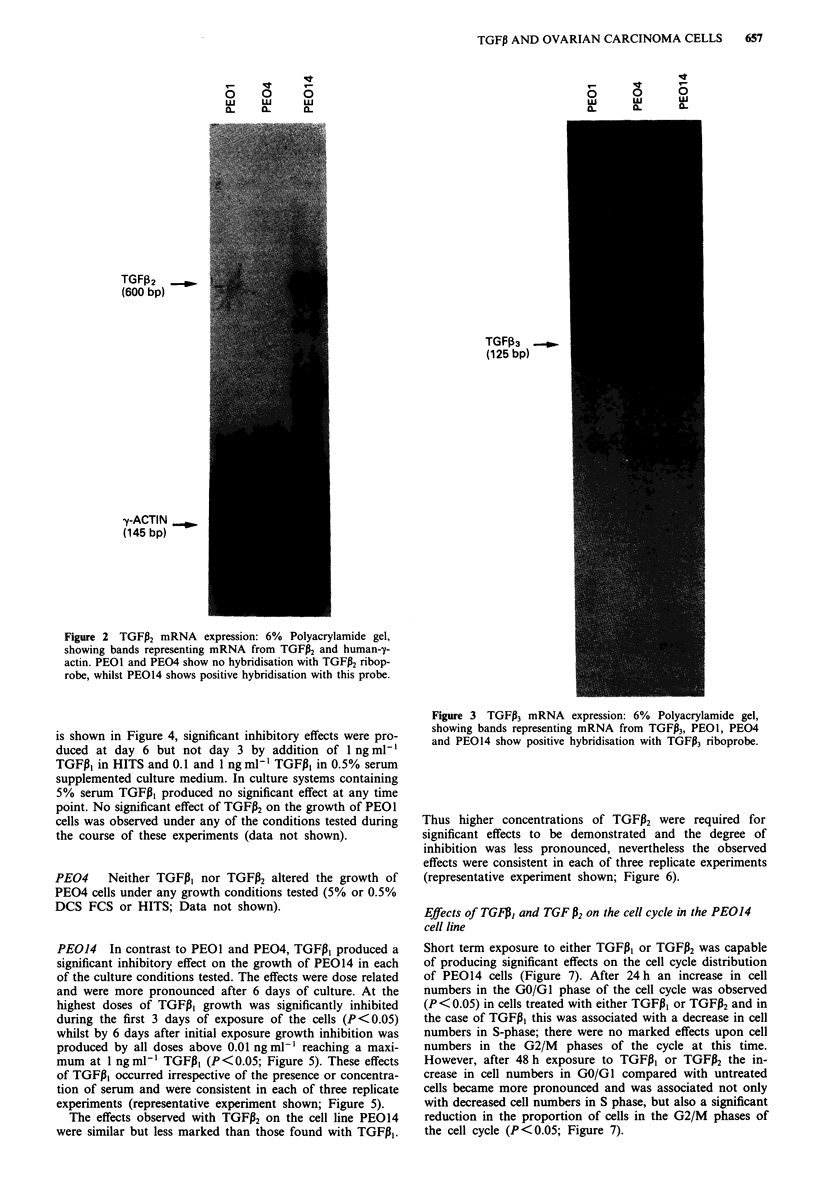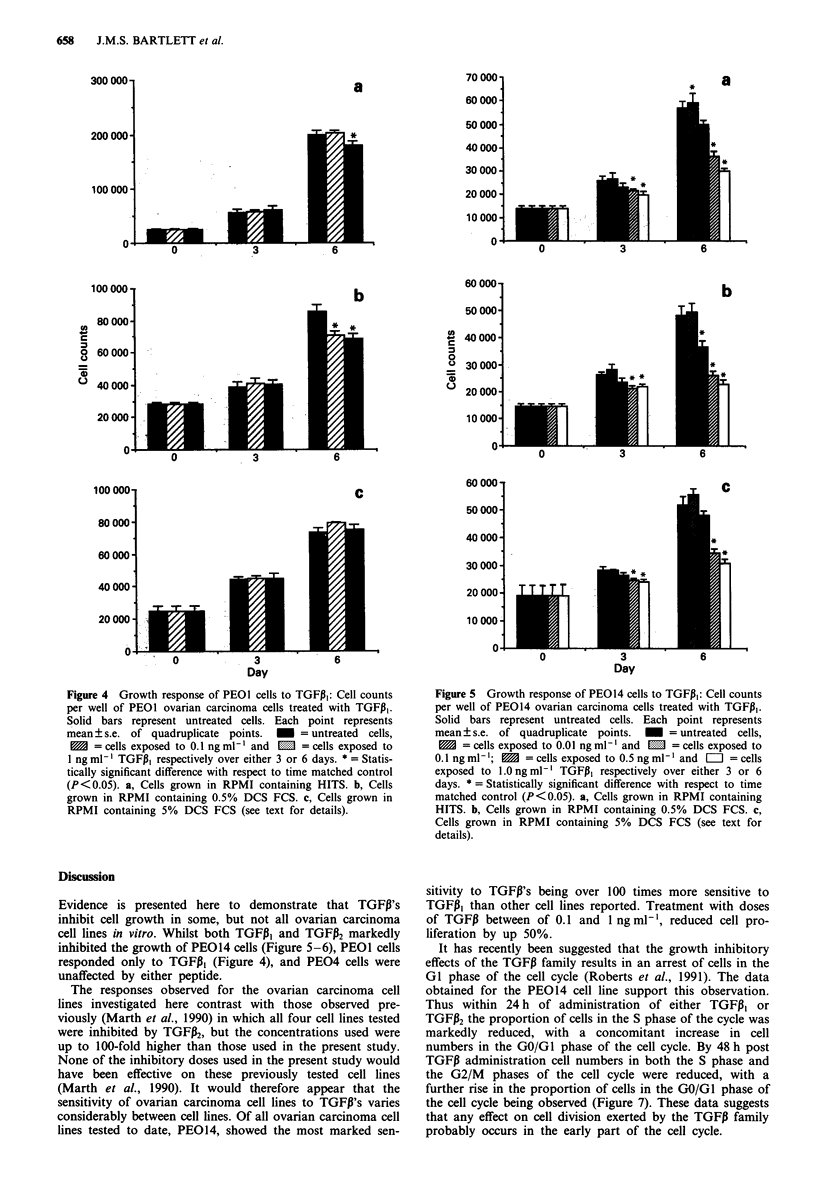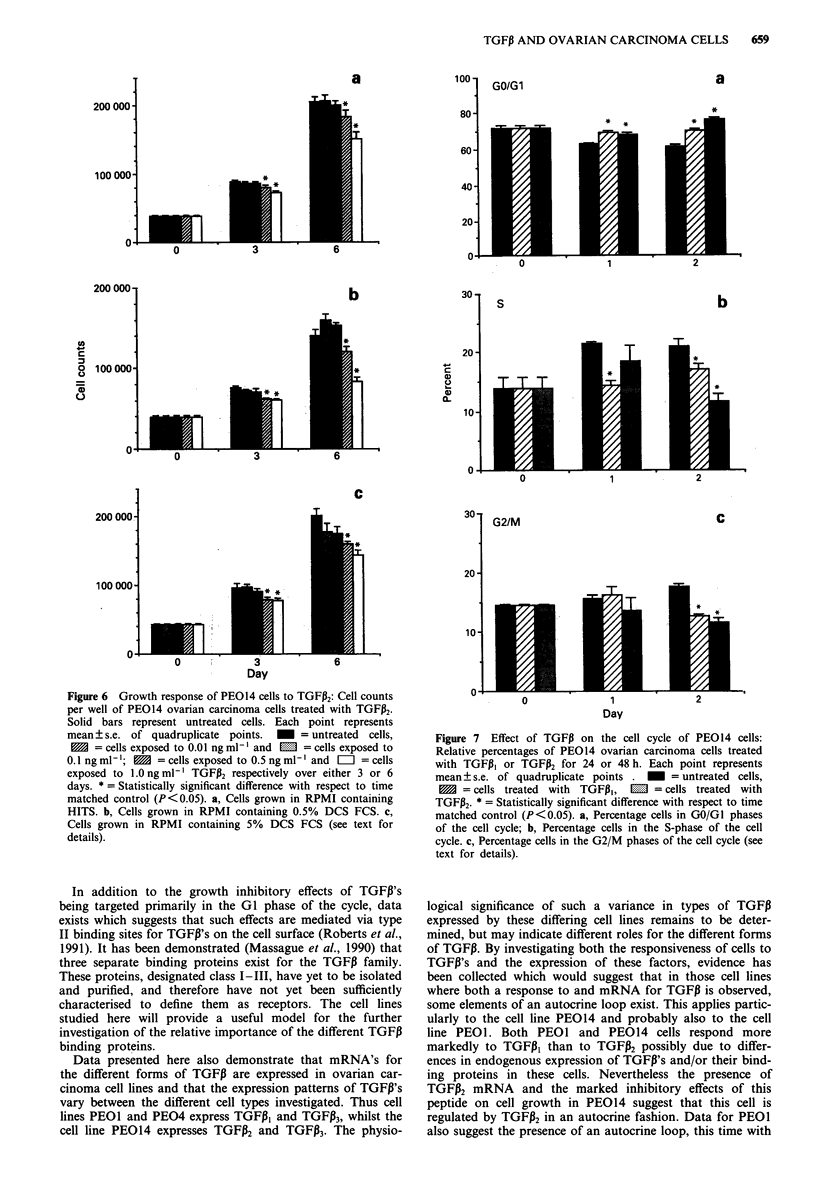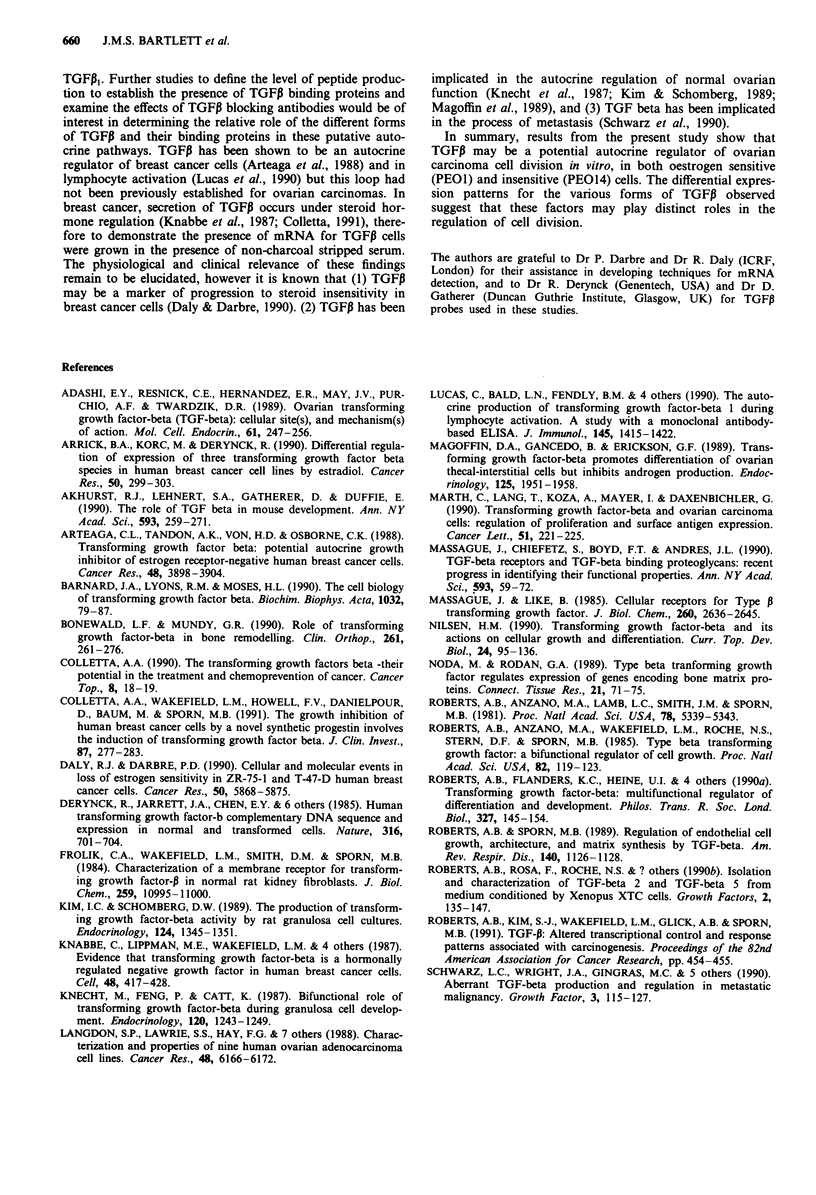# Transforming growth factor-beta mRNA expression and growth control of human ovarian carcinoma cells.

**DOI:** 10.1038/bjc.1992.140

**Published:** 1992-05

**Authors:** J. M. Bartlett, G. J. Rabiasz, W. N. Scott, S. P. Langdon, J. F. Smyth, W. R. Miller

**Affiliations:** ICRF Medical Oncology Unit, Western General Hospital, Edinburgh, UK.

## Abstract

**Images:**


*Br. J. Cancer* (1992), **65**, 655–660 © Macmillan Press Ltd., 1992

### Transforming growth factor-β mRNA expression and growth control of human ovarian carcinoma cells

J.M.S. Bartlett, G.J. Rabiasz, W.N. Scott, S.P. Langdon, J.F. Smyth & W.R. Miller

*ICRF Medical Oncology Unit, Western General Hospital, Edinburgh EH4 2XU, UK.*

**Summary** The pattern of TGFβ expression and *in vitro* response to TGFβ has been defined in three ovarian carcinoma cell lines (PEO1, PEO4 and PEO14). Marked differences in both mRNA expression and growth responses were detected between the cell lines. All expressed mRNA for $TGF\beta_3$, PEO1 and PEO4 but not PEO14 expressed mRNA for $TGF\beta_1$, whereas PEO14 but not PEO1 and PEO4 expressed $TGF\beta_2$. Growth of PEO14 cells in culture was markedly inhibited by both $TGF\beta_1$ and $\beta_2$, PEO1 cells were inhibited by $TGF\beta_1$, but not $TGF\beta_2$ whilst growth of PEO4 cells were not affected by exposure to either of these peptides. These data indicate that several elements of potential autocrine loops involving TGFβ's are present within ovarian cancer cells.

The transforming growth factors are increasingly recognised as important molecules in the regulation of cell growth and differentiation (Nilsen, 1990; Barnard *et al.*, 1990 for reviews). Whilst the role of TGF-α has been relatively widely studied, data on the TGFβ's are only now becoming available. These peptides are generating interest in a variety of fields including development (Akhurst *et al.*, 1990; Roberts *et al.*, 1990*a*), bone remodelling (Noda & Rodan, 1989; Bonewald & Mundy, 1990), extracellular matrix production (Roberts & Sporn, 1989) and the prevention and treatment of cancer (Colletta, 1990). Research into the biological role of TGFβ's is complicated by the diversity of the peptide family, with at least three TGF-β peptides being identified thus far in the human (Derynck *et al.*, 1985; Arrick *et al.*, 1990) and additional forms being present in other species (Roberts *et al.*, 1990*b*). However, TGFβ has been detected in a wide range of tissues, including transformed cells (Roberts *et al.*, 1981), and dependent upon conditions it may be either stimulatory or inhibitory for cell growth (Roberts *et al.*, 1985). There are multiple binding proteins for TGFβ's (Frolik *et al.*, 1984; Massague & Like, 1985; Massague *et al.*, 1990) and recent data suggest that the growth regulatory effects of TGFβ may be dependent upon the presence of specific classes of binding proteins (Roberts, 1991).

TGFβ appears to play a role in normal ovarian function, particularly in the regulation of granulosa cell functions in response to follicle stimulating hormone (Adashi *et al.*, 1989). However, little is known of the effects of TGFβ's and their expression in ovarian carcinomas. In order to investigate further the role of TGFβ in ovarian carcinoma cells, we have developed cell line models and have examined them for the presence of TGFβ mRNA and their growth response to TGF-β.

### Materials and methods

### Cell lines

The human ovarian carcinoma cell lines PEO1, PEO4 and PEO14 were established and characterised as previously described (Langdon *et al.*, 1988). They were maintained routinely at 37°C in a humidified atmosphere of 5% $CO_2$ in air in RPMI 1640 (Gibco) supplemented with Streptomycin (100 $\mu g\ ml^{-1}$), Penicillin (100 IU $ml^{-1}$) and glutamine (2 mM; 'RPMI') and containing 5% non-charcoal stripped heat-inactivated foetal calf serum (FCS).

### *Effects of $TGF\beta_1$ and TGF $\beta_2$ on growth of ovarian carcinoma cell lines*

Exponentially growing cells were harvested by trypsinisation and plated in 24-well plates (Falcon) at densities of approximately $2 \times 10^4$ cells/well (four wells per experimental condition) in RPMI containing 5% FCS. After 24 h, medium was removed, cells were washed with phosphate buffered saline (pH 7.4; PBS) and medium replaced with RPMI containing either 5% double charcoal stripped (DCS), FCS, 0.5% DCS, FCS or HITS (hydrocortisone 10 nm, insulin 5 $\mu g\ ml^{-1}$, transferrin 10 $\mu g\ ml^{-1}$, selenium 30 nm) and incubated for a further 24 h. Cells were then washed with PBS and medium replaced with RPMI with the corresponding additives (as above) with or without human recombinant TGF-$\beta_1$ or porcine TGF-$\beta_2$ (British Biotechnology) added at concentrations ranging from 0.01 ng $ml^{-1}$ to 1.0 ng $ml^{-1}$. This time point was designated day 0. Cells were incubated at 37°C for 6 days. Media was replenished on day 3. On days 0, 3, and 6, cells were harvested by trypsinisation and counted using a Coulter Counter.

### *Effects of $TGF\beta_2$ and TGF $\beta_2$ on the cell cycle in the PEO14 cell line*

Exponentially growing cells were harvested by trypsinisation and plated in 6-well plates (Falcon) at densities of approximately $2 \times 10^4$ cells well (four wells per experimental condition) in RPMI containing 5% FCS . After 24 h, medium was removed, cells were washed with PBS and medium replaced with RPMI containing 5% DCS, FCS with or without added growth factor. Cells were harvested by trypsinisation at time 0 and after 24 and 48 h incubation with $TGF\beta_1$ or $TGF\beta_2$ at a concentration of 1 ng $ml^{-1}$. The cell suspension was transferred to plastic tubes containing 0.5 ml FCS (to neutralise trypsin) and cells centrifuged for 4 min at 500 *g* at room temperature, washed in PBS and repelleted. Ethanol (0.5 ml 70%) was added to the cells and pellets stored at −40°C until required for analysis. Cells were treated with detergent and the DNA stained with propidium iodide (Vindelov *et al.*, 1983). Analysis was performed using a FACScan flow cytometer (Becton Dickinson), with gates set to exclude fragmented or clumped material and doublets.

### *mRNA extraction*

Exponentially growing cells were harvested from 175 $cm^2$ culture flasks as follows: Cells were washed with ice cold

Correspondence: J.M.S. Bartlett, ICRF Medical Oncology Unit, Western General Hospital, Crewe Road, Edinburgh EH4 2XU, UK.
Received 10 October 1991; and in revised form 20 December 1991.

PBS, harvested using a cell scraper, suspended in ice cold PBS (25 ml) and spun down in a bench top centrifuge (1,000 *g*, 10 min). The cell pellet was stored at −70°C until used for RNA extraction. Using a sterile pasteur pipette the cell pellet was transferred to a 15 ml tube containing 3 M lithium chloride/6 M urea (6 ml). The homogenate was sonicated twice at 4°C for 30 s and stored overnight at 4°C. The pellet was spun down at 15,000 *g*, 4°C for 30 min. The supernatant was discarded and the pellet washed with fresh lithium chloride/urea (6 ml) and centrifuged at 15,000 *g*, 4°C for 30 min. The pellet was then resuspended in Tris-HCl (10 mM pH 7.5, 6 ml) SDS (0.5%), with proteinase K (50 μg $ml^{-1}$, Boehringer Mannheim) added and the sample incubated at 37°C for 20 min. Following incubation the samples were extracted using 100% phenol (pre-equilibrated with 0.1 M Tris pH 7.4), this extraction was repeated using phenol:chloroform: isoamyl-alcohol (25:24:1 v/v/v) and chloroform:isoamyl-alcohol (24:1 v/v). Following each extraction the sample was centrifuged at 2,000 *g* at room temperature for 10 min and the aqueous phase recovered. After the final extraction, lithium chloride (300 μl 8 M), absolute alcohol (2.5 volumes) were added and the RNA precipitated overnight at −20°C. RNA was pelleted by centrifugation at 4,000 *g*, 4°C for 45 min. The supernatant was decanted and the pellet dried and resuspended in diethylpyrocarbonate treated water. Optical density measurements at 260 and 280 nm were taken to assess yield and purity of the RNA preparation.

*Synthesis of riboprobes*

Labelled RNA was prepared from linearised template DNA using a Gemini II system (Promega Ltd, Southampton, UK). Template DNA was incubated in the presence of an RNAase inhibitor (Human placental RNAsin; Amersham plc), cold ribonucleosides, dithiothreitol and $^{32}$P-rCTP with the appropriate RNA polymerase (T3, T7 or SP6) for 1 h at 37°C. The DNA template was then removed by incubation with RQ1 DNAase (Promega Ltd) for 15 min at 37°C. Labelled RNA was precipitated in the presence of added tRNA (Sigma) as carrier and full length transcripts were isolated by polyacrylamide electrophoresis. Following identification of full length transcripts by autoradiography, the bands were excised and labelled RNA eluted from the gel, precipitated under ethanol and resuspended in hybridisation buffer prior to use in RNAase protection assays.

*RNAase protection assay*

Test RNA (20 μg) was precipitated under ethanol, dried and resuspended in 30 μl hybridisation buffer (80% formamide, 40 mM Pipes (pH 6.7), 400 mM NaCl, 1 mM EDTA); tRNA was prepared in a similar manner as a negative control. Test probe ($10^6$ c.p.m.) plus actin probe ($10^6$ c.p.m.) were added to each sample. Samples were incubated at 85°C for 20 min, transferred to a water bath and left to hybridise overnight at 51°C. After hybridisation, single stranded RNA (both labelled and cold) was removed by incubating with single strand specific RNAases A and T1 (Boehringer Mannheim) at 37°C for 30 min, followed by incubation with proteinase K in SDS at 37°C for 15 min. Protein was extracted by using phenol/chloroform-isoamyl alcohol. Double stranded probe: test RNA was precipitated with carrier tRNA (5 μg) and separated by gel electrophoresis. Full length transcripts for test probes were scored as positive, whilst transcripts for actin were used as an internal control.

*Statistics*

Cell growth and cell cycle responses *in vitro* were analysed using Wilcoxon Rank test and significant differences at the $P < 0.05$ level defined.

### Results

*TGFβ mRNA expression in ovarian carcinoma cell lines*

TGF$\beta_1$ mRNA expression was observed only in cell lines PEO1 and PEO4, and not in PEO14 cells (Figure 1). In contrast, expression of mRNA for TGF$\beta_2$ could not be demonstrated in PEO1 or PEO4 cell extracts whilst PEO14 cells appeared to express this factor (Figure 2). Finally, TGF$\beta_3$ mRNA expression was observed in all three cell lines tested (Figure 3).

*Growth responsiveness of ovarian carcinoma cell lines to TGF$\beta_1$ and TGF$\beta_2$*

*PEO1* TGF$\beta_1$ was capable of producing significant inhibitory effects on the growth of PEO1 cells, but these were small and observed only under certain culture conditions. Thus, as

**Figure 1** TGF$\beta_1$ mRNA expression: 6% Polyacrylamide gel, showing bands representing mRNA from TGF$\beta_1$ and human-γ-actin. Lanes 1–3 contain test samples, PEO1 and PEO4 show positive hybridisation with TGF$\beta_1$ riboprobe, whilst no signal is apparent with PEO14 mRNA. Lane 4 contains tRNA as a negative control.

**Figure 2** $TGF\beta_2$ mRNA expression: 6% Polyacrylamide gel, showing bands representing mRNA from $TGF\beta_2$ and human-γ-actin. PEO1 and PEO4 show no hybridisation with $TGF\beta_2$ riboprobe, whilst PEO14 shows positive hybridisation with this probe.

**Figure 3** $TGF\beta_3$ mRNA expression: 6% Polyacrylamide gel, showing bands representing mRNA from $TGF\beta_3$, PEO1, PEO4 and PEO14 show positive hybridisation with $TGF\beta_3$ riboprobe.

is shown in Figure 4, significant inhibitory effects were produced at day 6 but not day 3 by addition of 1 ng ml$^{-1}$ $TGF\beta_1$ in HITS and 0.1 and 1 ng ml$^{-1}$ $TGF\beta_1$ in 0.5% serum supplemented culture medium. In culture systems containing 5% serum $TGF\beta_1$ produced no significant effect at any time point. No significant effect of $TGF\beta_2$ on the growth of PEO1 cells was observed under any of the conditions tested during the course of these experiments (data not shown).

*PEO4* Neither $TGF\beta_1$ nor $TGF\beta_2$ altered the growth of PEO4 cells under any growth conditions tested (5% or 0.5% DCS FCS or HITS; Data not shown).

*PEO14* In contrast to PEO1 and PEO4, $TGF\beta_1$ produced a significant inhibitory effect on the growth of PEO14 in each of the culture conditions tested. The effects were dose related and were more pronounced after 6 days of culture. At the highest doses of $TGF\beta_1$ growth was significantly inhibited during the first 3 days of exposure of the cells ($P<0.05$) whilst by 6 days after initial exposure growth inhibition was produced by all doses above 0.01 ng ml$^{-1}$ reaching a maximum at 1 ng ml$^{-1}$ $TGF\beta_1$ ($P<0.05$; Figure 5). These effects of $TGF\beta_1$ occurred irrespective of the presence or concentration of serum and were consistent in each of three replicate experiments (representative experiment shown; Figure 5).

The effects observed with $TGF\beta_2$ on the cell line PEO14 were similar but less marked than those found with $TGF\beta_1$. Thus higher concentrations of $TGF\beta_2$ were required for significant effects to be demonstrated and the degree of inhibition was less pronounced, nevertheless the observed effects were consistent in each of three replicate experiments (representative experiment shown; Figure 6).

### *Effects of $TGF\beta_1$ and $TGF\ \beta_2$ on the cell cycle in the PEO14 cell line*

Short term exposure to either $TGF\beta_1$ or $TGF\beta_2$ was capable of producing significant effects on the cell cycle distribution of PEO14 cells (Figure 7). After 24 h an increase in cell numbers in the G0/G1 phase of the cell cycle was observed ($P<0.05$) in cells treated with either $TGF\beta_1$ or $TGF\beta_2$ and in the case of $TGF\beta_1$ this was associated with a decrease in cell numbers in S-phase; there were no marked effects upon cell numbers in the G2/M phases of the cycle at this time. However, after 48 h exposure to $TGF\beta_1$ or $TGF\beta_2$ the increase in cell numbers in G0/G1 compared with untreated cells became more pronounced and was associated not only with decreased cell numbers in S phase, but also a significant reduction in the proportion of cells in the G2/M phases of the cell cycle ($P<0.05$; Figure 7).

**Figure 4** Growth response of PEO1 cells to TGF$\beta_1$: Cell counts per well of PEO1 ovarian carcinoma cells treated with TGF$\beta_1$. Solid bars represent untreated cells. Each point represents mean ± s.e. of quadruplicate points. ■ = untreated cells, ▨ = cells exposed to 0.1 ng ml$^{-1}$ and ▩ = cells exposed to 1 ng ml$^{-1}$ TGF$\beta_1$ respectively over either 3 or 6 days. * = Statistically significant difference with respect to time matched control ($P<0.05$). **a**, Cells grown in RPMI containing HITS. **b**, Cells grown in RPMI containing 0.5% DCS FCS. **c**, Cells grown in RPMI containing 5% DCS FCS (see text for details).

**Figure 5** Growth response of PEO14 cells to TGF$\beta_1$: Cell counts per well of PEO14 ovarian carcinoma cells treated with TGF$\beta_1$. Solid bars represent untreated cells. Each point represents mean ± s.e. of quadruplicate points. ■ = untreated cells, ▨ = cells exposed to 0.01 ng ml$^{-1}$ and ▩ = cells exposed to 0.1 ng ml$^{-1}$; ▨ = cells exposed to 0.5 ng ml$^{-1}$ and □ = cells exposed to 1.0 ng ml$^{-1}$ TGF$\beta_1$ respectively over either 3 or 6 days. * = Statistically significant difference with respect to time matched control ($P<0.05$). **a**, Cells grown in RPMI containing HITS. **b**, Cells grown in RPMI containing 0.5% DCS FCS. **c**, Cells grown in RPMI containing 5% DCS FCS (see text for details).

### Discussion

Evidence is presented here to demonstrate that TGF$\beta$'s inhibit cell growth in some, but not all ovarian carcinoma cell lines *in vitro*. Whilst both TGF$\beta_1$ and TGF$\beta_2$ markedly inhibited the growth of PEO14 cells (Figure 5–6), PEO1 cells responded only to TGF$\beta_1$ (Figure 4), and PEO4 cells were unaffected by either peptide.

The responses observed for the ovarian carcinoma cell lines investigated here contrast with those observed previously (Marth *et al.*, 1990) in which all four cell lines tested were inhibited by TGF$\beta_2$, but the concentrations used were up to 100-fold higher than those used in the present study. None of the inhibitory doses used in the present study would have been effective on these previously tested cell lines (Marth *et al.*, 1990). It would therefore appear that the sensitivity of ovarian carcinoma cell lines to TGF$\beta$'s varies considerably between cell lines. Of all ovarian carcinoma cell lines tested to date, PEO14, showed the most marked sensitivity to TGF$\beta$'s being over 100 times more sensitive to TGF$\beta_1$ than other cell lines reported. Treatment with doses of TGF$\beta$ between of 0.1 and 1 ng ml$^{-1}$, reduced cell proliferation by up 50%.

It has recently been suggested that the growth inhibitory effects of the TGF$\beta$ family results in an arrest of cells in the G1 phase of the cell cycle (Roberts *et al.*, 1991). The data obtained for the PEO14 cell line support this observation. Thus within 24 h of administration of either TGF$\beta_1$ or TGF$\beta_2$ the proportion of cells in the S phase of the cycle was markedly reduced, with a concomitant increase in cell numbers in the G0/G1 phase of the cell cycle. By 48 h post TGF$\beta$ administration cell numbers in both the S phase and the G2/M phases of the cell cycle were reduced, with a further rise in the proportion of cells in the G0/G1 phase of the cell cycle being observed (Figure 7). These data suggests that any effect on cell division exerted by the TGF$\beta$ family probably occurs in the early part of the cell cycle.

**Figure 6** Growth response of PEO14 cells to TGF$\beta_2$: Cell counts per well of PEO14 ovarian carcinoma cells treated with TGF$\beta_2$. Solid bars represent untreated cells. Each point represents mean ± s.e. of quadruplicate points. ■ = untreated cells, ▨ = cells exposed to 0.01 ng ml$^{-1}$ and ▩ = cells exposed to 0.1 ng ml$^{-1}$; ▨ = cells exposed to 0.5 ng ml$^{-1}$ and □ = cells exposed to 1.0 ng ml$^{-1}$ TGF$\beta_2$ respectively over either 3 or 6 days. * = Statistically significant difference with respect to time matched control ($P < 0.05$). **a**, Cells grown in RPMI containing HITS. **b**, Cells grown in RPMI containing 0.5% DCS FCS. **c**, Cells grown in RPMI containing 5% DCS FCS (see text for details).

**Figure 7** Effect of TGFβ on the cell cycle of PEO14 cells: Relative percentages of PEO14 ovarian carcinoma cells treated with TGF$\beta_1$ or TGF$\beta_2$ for 24 or 48 h. Each point represents mean ± s.e. of quadruplicate points . ■ = untreated cells, ▨ = cells treated with TGF$\beta_1$, ▩ = cells treated with TGF$\beta_2$. * = Statistically significant difference with respect to time matched control ($P < 0.05$). **a**, Percentage cells in G0/G1 phases of the cell cycle; **b**, Percentage cells in the S-phase of the cell cycle. **c**, Percentage cells in the G2/M phases of the cell cycle (see text for details).

In addition to the growth inhibitory effects of TGFβ's being targeted primarily in the G1 phase of the cycle, data exists which suggests that such effects are mediated via type II binding sites for TGFβ's on the cell surface (Roberts *et al.*, 1991). It has been demonstrated (Massague *et al.*, 1990) that three separate binding proteins exist for the TGFβ family. These proteins, designated class I–III, have yet to be isolated and purified, and therefore have not yet been sufficiently characterised to define them as receptors. The cell lines studied here will provide a useful model for the further investigation of the relative importance of the different TGFβ binding proteins.

Data presented here also demonstrate that mRNA's for the different forms of TGFβ are expressed in ovarian carcinoma cell lines and that the expression patterns of TGFβ's vary between the different cell types investigated. Thus cell lines PEO1 and PEO4 express TGF$\beta_1$ and TGF$\beta_3$, whilst the cell line PEO14 expresses TGF$\beta_2$ and TGF$\beta_3$. The physiological significance of such a variance in types of TGFβ expressed by these differing cell lines remains to be determined, but may indicate different roles for the different forms of TGFβ. By investigating both the responsiveness of cells to TGFβ's and the expression of these factors, evidence has been collected which would suggest that in those cell lines where both a response to and mRNA for TGFβ is observed, some elements of an autocrine loop exist. This applies particularly to the cell line PEO14 and probably also to the cell line PEO1. Both PEO1 and PEO14 cells respond more markedly to TGF$\beta_1$ than to TGF$\beta_2$ possibly due to differences in endogenous expression of TGFβ's and/or their binding proteins in these cells. Nevertheless the presence of TGF$\beta_2$ mRNA and the marked inhibitory effects of this peptide on cell growth in PEO14 suggest that this cell is regulated by TGF$\beta_2$ in an autocrine fashion. Data for PEO1 also suggest the presence of an autocrine loop, this time with

$TGF\beta_1$. Further studies to define the level of peptide production to establish the presence of TGFβ binding proteins and examine the effects of TGFβ blocking antibodies would be of interest in determining the relative role of the different forms of TGFβ and their binding proteins in these putative autocrine pathways. TGFβ has been shown to be an autocrine regulator of breast cancer cells (Arteaga *et al.*, 1988) and in lymphocyte activation (Lucas *et al.*, 1990) but this loop had not been previously established for ovarian carcinomas. In breast cancer, secretion of TGFβ occurs under steroid hormone regulation (Knabbe *et al.*, 1987; Colletta, 1991), therefore to demonstrate the presence of mRNA for TGFβ cells were grown in the presence of non-charcoal stripped serum. The physiological and clinical relevance of these findings remain to be elucidated, however it is known that (1) TGFβ may be a marker of progression to steroid insensitivity in breast cancer cells (Daly & Darbre, 1990). (2) TGFβ has been implicated in the autocrine regulation of normal ovarian function (Knecht *et al.*, 1987; Kim & Schomberg, 1989; Magoffin *et al.*, 1989), and (3) TGF beta has been implicated in the process of metastasis (Schwarz *et al.*, 1990).

In summary, results from the present study show that TGFβ may be a potential autocrine regulator of ovarian carcinoma cell division *in vitro*, in both oestrogen sensitive (PEO1) and insensitive (PEO14) cells. The differential expression patterns for the various forms of TGFβ observed suggest that these factors may play distinct roles in the regulation of cell division.

The authors are grateful to Dr P. Darbre and Dr R. Daly (ICRF, London) for their assistance in developing techniques for mRNA detection, and to Dr R. Derynck (Genentech, USA) and Dr D. Gatherer (Duncan Guthrie Institute, Glasgow, UK) for TGFβ probes used in these studies.

### References

ADASHI, E.Y., RESNICK, C.E., HERNANDEZ, E.R., MAY, J.V., PURCHIO, A.F. & TWARDZIK, D.R. (1989). Ovarian transforming growth factor-beta (TGF-beta): cellular site(s), and mechanism(s) of action. *Mol. Cell. Endocrin.*, **61**, 247–256.

ARRICK, B.A., KORC, M. & DERYNCK, R. (1990). Differential regulation of expression of three transforming growth factor beta species in human breast cancer cell lines by estradiol. *Cancer Res.*, **50**, 299–303.

AKHURST, R.J., LEHNERT, S.A., GATHERER, D. & DUFFIE, E. (1990). The role of TGF beta in mouse development. *Ann. NY Acad. Sci.*, **593**, 259–271.

ARTEAGA, C.L., TANDON, A.K., VON, H.D. & OSBORNE, C.K. (1988). Transforming growth factor beta: potential autocrine growth inhibitor of estrogen receptor-negative human breast cancer cells. *Cancer Res.*, **48**, 3898–3904.

BARNARD, J.A., LYONS, R.M. & MOSES, H.L. (1990). The cell biology of transforming growth factor beta. *Biochim. Biophys. Acta*, **1032**, 79–87.

BONEWALD, L.F. & MUNDY, G.R. (1990). Role of transforming growth factor-beta in bone remodelling. *Clin. Orthop.*, **261**, 261–276.

COLLETTA, A.A. (1990). The transforming growth factors beta -their potential in the treatment and chemoprevention of cancer. *Cancer Top.*, **8**, 18–19.

COLLETTA, A.A., WAKEFIELD, L.M., HOWELL, F.V., DANIELPOUR, D., BAUM, M. & SPORN, M.B. (1991). The growth inhibition of human breast cancer cells by a novel synthetic progestin involves the induction of transforming growth factor beta. *J. Clin. Invest.*, **87**, 277–283.

DALY, R.J. & DARBRE, P.D. (1990). Cellular and molecular events in loss of estrogen sensitivity in ZR-75-1 and T-47-D human breast cancer cells. *Cancer Res.*, **50**, 5868–5875.

DERYNCK, R., JARRETT, J.A., CHEN, E.Y. & 6 others (1985). Human transforming growth factor-b complementary DNA sequence and expression in normal and transformed cells. *Nature*, **316**, 701–704.

FROLIK, C.A., WAKEFIELD, L.M., SMITH, D.M. & SPORN, M.B. (1984). Characterization of a membrane receptor for transforming growth factor-β in normal rat kidney fibroblasts. *J. Biol. Chem.*, **259**, 10995–11000.

KIM, I.C. & SCHOMBERG, D.W. (1989). The production of transforming growth factor-beta activity by rat granulosa cell cultures. *Endocrinology*, **124**, 1345–1351.

KNABBE, C., LIPPMAN, M.E., WAKEFIELD, L.M. & 4 others (1987). Evidence that transforming growth factor-beta is a hormonally regulated negative growth factor in human breast cancer cells. *Cell*, **48**, 417–428.

KNECHT, M., FENG, P. & CATT, K. (1987). Bifunctional role of transforming growth factor-beta during granulosa cell development. *Endocrinology*, **120**, 1243–1249.

LANGDON, S.P., LAWRIE, S.S., HAY, F.G. & 7 others (1988). Characterization and properties of nine human ovarian adenocarcinoma cell lines. *Cancer Res.*, **48**, 6166–6172.

LUCAS, C., BALD, L.N., FENDLY, B.M. & 4 others (1990). The autocrine production of transforming growth factor-beta 1 during lymphocyte activation. A study with a monoclonal antibody-based ELISA. *J. Immunol.*, **145**, 1415–1422.

MAGOFFIN, D.A., GANCEDO, B. & ERICKSON, G.F. (1989). Transforming growth factor-beta promotes differentiation of ovarian thecal-interstitial cells but inhibits androgen production. *Endocrinology*, **125**, 1951–1958.

MARTH, C., LANG, T., KOZA, A., MAYER, I. & DAXENBICHLER, G. (1990). Transforming growth factor-beta and ovarian carcinoma cells: regulation of proliferation and surface antigen expression. *Cancer Lett.*, **51**, 221–225.

MASSAGUE, J., CHIEFETZ, S., BOYD, F.T. & ANDRES, J.L. (1990). TGF-beta receptors and TGF-beta binding proteoglycans: recent progress in identifying their functional properties. *Ann. NY Acad. Sci.*, **593**, 59–72.

MASSAGUE, J. & LIKE, B. (1985). Cellular receptors for Type β transforming growth factor. *J. Biol. Chem.*, **260**, 2636–2645.

NILSEN, H.M. (1990). Transforming growth factor-beta and its actions on cellular growth and differentiation. *Curr. Top. Dev. Biol.*, **24**, 95–136.

NODA, M. & RODAN, G.A. (1989). Type beta tranforming growth factor regulates expression of genes encoding bone matrix proteins. *Connect. Tissue Res.*, **21**, 71–75.

ROBERTS, A.B., ANZANO, M.A., LAMB, L.C., SMITH, J.M. & SPORN, M.B. (1981). *Proc. Natl Acad. Sci. USA*, **78**, 5339–5343.

ROBERTS, A.B., ANZANO, M.A., WAKEFIELD, L.M., ROCHE, N.S., STERN, D.F. & SPORN, M.B. (1985). Type beta transforming growth factor: a bifunctional regulator of cell growth. *Proc. Natl Acad. Sci. USA*, **82**, 119–123.

ROBERTS, A.B., FLANDERS, K.C., HEINE, U.I. & 4 others (1990*a*). Transforming growth factor-beta: multifunctional regulator of differentiation and development. *Philos. Trans. R. Soc. Lond. Biol.*, **327**, 145–154.

ROBERTS, A.B. & SPORN, M.B. (1989). Regulation of endothelial cell growth, architecture, and matrix synthesis by TGF-beta. *Am. Rev. Respir. Dis.*, **140**, 1126–1128.

ROBERTS, A.B., ROSA, F., ROCHE, N.S. & ? others (1990*b*). Isolation and characterization of TGF-beta 2 and TGF-beta 5 from medium conditioned by Xenopus XTC cells. *Growth Factors*, **2**, 135–147.

ROBERTS, A.B., KIM, S.-J., WAKEFIELD, L.M., GLICK, A.B. & SPORN, M.B. (1991). TGF-β: Altered transcriptional control and response patterns associated with carcinogenesis. *Proceedings of the 82nd American Association for Cancer Research*, pp. 454–455.

SCHWARZ, L.C., WRIGHT, J.A., GINGRAS, M.C. & 5 others (1990). Aberrant TGF-beta production and regulation in metastatic malignancy. *Growth Factor*, **3**, 115–127.